# Formulation and Evaluation of Liquisolid Compacts for Olmesartan Medoxomil

**DOI:** 10.1155/2013/870579

**Published:** 2013-10-21

**Authors:** Shailesh T. Prajapati, Hitesh H. Bulchandani, Dashrath M. Patel, Suresh K. Dumaniya, Chhaganbhai N. Patel

**Affiliations:** ^1^Department of Pharmaceutics, Shri Sarvajanik Pharmacy College, Near Arvind Baug, Mehsana, Gujarat 384001, India; ^2^Department of Pharmaceutical Chemistry, Shri Sarvajanik Pharmacy College, Near Arvind Baug, Mehsana, Gujarat 384001, India

## Abstract

Olmesartan medoxomil is an angiotensin type II receptor blocker, antihypertensive agent, administered orally. It is highly lipophilic (log *P* 5.5) and a poorly water-soluble drug with absolute bioavailability of 26%. The poor dissolution rate of water-insoluble drugs is still a major problem confronting the pharmaceutical industry. The objective of the present investigation was to develop liquisolid compacts for olmesartan medoxomil to improve the dissolution rate. Liquisolid compacts were prepared using Acrysol El 135 as a solvent, Avicel PH 102, Fujicalin and Neusilin as carrier materials, and Aerosil as coating material in different ratios. The interaction between drug and excipients was characterized by DSC and FT-IR studies, which showed that there is no interaction between drug and excipients. The powder characteristics were evaluated by different flow parameters to comply with pharmacopoeial limits. The dissolution studies for liquisolid compacts and conventional formulations were carried out, and it was found that liquisolid compacts with 80% w/w of Acrysol EL 135 to the drug showed significant higher drug release rates than conventional tablets. Amongst carriers used Fujicalin and Neusilin were found to be more effective carrier materials for liquid adsorption.

## 1. Introduction

As a most discussed but still not completely resolved issue, solubility or dissolution enhancement techniques remain the most vibrant field for the researchers in formulation science. Solubility and dissolution are the core concepts of any physical or chemical science including biopharmaceutical and pharmacokinetic considerations in therapy of any medicine. The solubility/dissolution behavior of a drug is key determinant to its oral bioavailability, the latest frequency being the rate-limiting step of absorption of drugs from the gastrointestinal tract. As a result, more than 40% of new candidates entering drug development pipeline fail because of nonoptimal biopharmaceutical properties [1].

**Figure 1:**
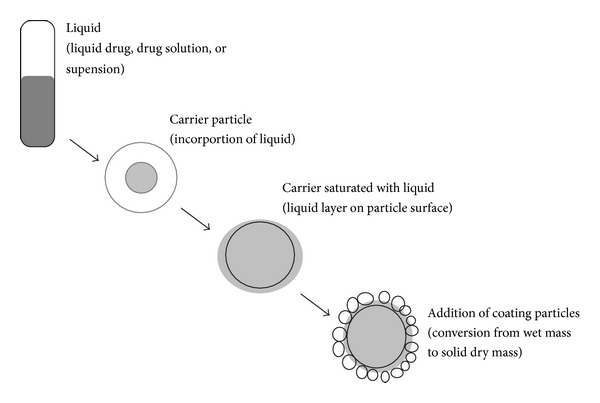
Schematic representation of liquisolid systems.

Over the years, various techniques have been employed to enhance the dissolution profile and, in turn, the absorption efficiency and bioavailability of water insoluble drugs and/or liquid lipophilic medication [2]. Several researchers have shown that the liquisolid technique is the most promising method for promoting dissolution rate of poorly water-soluble drugs [3–5]. The liquisolid technology is described by Spireas as liquid may be transformed into a free-flowing, readily compressible, and apparently dry powder by simple physical blending with selected excipients named the carrier and coating material (Figure 1). A liquid lipophilic drug can be converted into liquisolid system without being further modified. On the other hand, if a solid water-insoluble drug is formulated, it should be initially dissolved or suspended in suitable nonvolatile solvent system to produce drug solution or drug suspension of desired concentration. Inert, preferably water-miscible organic solvent systems with high boiling point and a not highly viscous organic solvent system such as propylene glycol, liquid polyethylene glycols, polysorbates, fixed oils, or glycerine are best suitable as liquid vehicles [5].

Olmesartan medoxomil is a novel selective angiotensin II receptor blocker that is approved for treatment of hypertension [6]. It is a prodrug rapidly deesterified during absorption from the gastrointestinal tract to produce an active metabolite, olmesartan [7]. However, the oral bioavailability of olmesartan medoxomil is only 26% in healthy humans due to low solubility in water and unfavorable breakage of the ester drug to a poorly permeable parent molecule in the gastrointestinal fluids. Olmesartan dose dependently reduces the blood pressure through arterial vasodilation and reduced sodium retention, as do other angiotensin receptor blockers [8].

Hence, the objective of the present work was to formulate the liquisolid compacts for olmesartan medoxomil to improve the solubility and dissolution rate, which can increase clinical efficacy or reduce the oral dosage required to achieve the same effect. 

## 2. Materials and Methods

### 2.1. Materials

Olmesartan medoxomil was received as a gift sample from Alembic Pharma Ltd., Baroda, India. The following materials were gifted by Abitec Corp., USA and were used as received: Capmul MCM (Glyceryl monocaprylate), Acconon C-80 (Polyoxyethylene 80 coconut glycerides), Captex 200 (Propylene glycol dicaprylocaprate), and Captex 355 (Glyceryl tricaprylate). Plurol Oleique (Polyglyceryl-3 dioleate), Labrafil M 2125CS (Linoleyl macrogol-6 glycerides), and Lauroglycol 90 (Propylene glycol monolaurate) were received as gift sample from Gattefosse, France. Acrysol K 140 (Polyoxyl 40 hydrogenated castor oil) and Acrysol EL 135 (Polyoxyl 35 castor oil) were procured as a gift samples from Corel Pharma Chem., Ahmedabad, India. Fujicalin (Dibasic calcium phosphate anhydrous) and Neusilin (Magnesium aluminometasilicate) were obtained as gift sample from Fuji Chemical Industry Co. Ltd., Japan.

### 2.2. Solubility Studies

Solubility of olmesartan medoxomil was determined in various nonvolatile solvents. Two mL of each component was taken in screw cap vials with known quantity (200 mg) of excess drug. After sealing, vials were kept on isothermal mechanical shaker at 37 ± 2°C for 72 hours. After equilibrium, each test tube was centrifuged at 6000 rpm for 20 minutes. Supernatant was filtered through membrane filter using 0.45 *μ*m filter disk. Filtered solution was appropriately diluted with methanol, and UV absorbances were measured at 257 nm wavelength. Concentration of dissolved drug was determined using standard equation.

### 2.3. Measuring Angle of Slide

This experiment was designed to measure the flowable liquid retention potential (*φ*-value) for Avicel PH 102, Fujicalin and Neusilin (carrier material, *φ*_Ca_), and Aerosil (coating material, *φ*_Co_) and the optimum liquid load factor (*L*_*f*_). The *φ*-value of a powder is the maximum amount of given nonvolatile liquid that can be retained inside powder bulk (w/w) while maintaining acceptable flowability, whereas *L*_*f*_ is the mass ratio (w/w) of the liquid medication to the carrier powder in the liquisolid formulation. Powder admixtures containing 5 g of either carrier or coating with increasing quantity of nonvolatile liquid vehicle (Acrysol EL 135) were mixed using a mortar and pestle. Each admixture was then placed on a shiny metal plate; the plate was then tilted until the admixture slides. The angle formed between the plate and the horizontal surface, at which admixture slides were measured as angle of slide (*θ*). The flowable liquid retention potential was calculated using the following equation:

(1) $$\begin{array}{c} \varphi \text{-Value}=\frac{\text{Weight}\;\text{of}\;\text{nonvolatile}\;\text{liquid}}{\text{Weight}\;\text{of}\;\text{carrier}\;\text{or}\;\text{coat}}. \end{array}$$

Each admixture has specific *φ*-values which were determined and plotted against respective measured angle of slide for all nonvolatile liquid vehicles. The *φ*-value that corresponds to an angle of slide of 33° was reported to represent the flowable liquid retention potentials of powder admixtures [9].

### 2.4. Preparation of Powder for Liquisolid and Conventional Tablets

Several olmesartan liquisolid formulations were prepared at two different drug concentrations of 20 and 40% (w/w) in liquid vehicles. Each formulation contains three different carriers, Avicel PH 102, Fujicalin and Neusilin and Aerosil as coating material at carrier/coat ratio of 5, 10, and 15. The appropriate amounts of carrier and coating materials used for each formulation depend upon *L*_*f*_of that formulation. The drug-vehicle liquid system was produced by mixing olmesartan (20 mg/tablet) in nonvolatile liquid vehicle using mortar and pestle. To this liquid medication, the calculated amount of the carrier was added by continuous mixing in the mortar. Then, coating material was carefully added and mixed until mortar contents start to look dry. In the last stage of the preparation, a 6% (w/w) croscarmellose as a disintegrant was added and mixed. All liquisolid preparations were compacted into tablets using a ten-station rotary compression machine (Rimek, Karnavati Engineering, India) using flat-faced punch with a compression force that provide acceptable tablet hardness. Composition of liquids solid compacts batches is shown in Table 1.

### 2.5. Precompression  Studies

Flowability  of  liquisolid admixture is important in formulation of tablet dosage form on industrial scale. Therefore, it was essential to study the flowability of these liquisolid powder admixtures prior to compression. Flowability can be evaluated using parameters such as Carr's index, angle of repose, and Hausner's ratio.

### 2.6. Angle of Repose

The angle of repose of powder blend was determined by fixed height funnel method. Angle of repose (*θ*) was calculated using the following equation:

(2) $$\begin{array}{c} \theta =\tan^{-1}\frac{\;h}{r}, \end{array}$$

where *h* and *r* are the height and radius of powder cone.

### 2.7. Compressibility Index

The compressibility index of the powder blend was determined by Carr's compressibility index [10]. The formula for Carr's index is as below:

(3) Carr's  index  (%)  =[(Tapped  density−Bulk  density)×100]Tapped  density.

### 2.8. Hausner's Ratio

Hausner's ratio was calculated from the equation:

(4) $$\begin{array}{c} \text{Hausner's}\;\text{ratio}=\frac{\text{Tapped}\;\text{density}}{\text{Bulk}\;\text{density}}. \end{array}$$

### 2.9. Differential Scanning Calorimetry

Differential Scanning Calorimetry study was carried out using calibrated Shimadzu DSC-60 (Shimadzu, Kyoto, Japan) instrument. DSC thermograms of pure drug olmesartan, and powder mixture for optimized liquisolid preparations were obtained. DSC aluminium cells were used as sample holder, and blank DSC aluminium cell was used as reference. 2-3 mg sample was used for analysis. Thermograms were recorded over the range of 20°C–300°C at a constant rate of 20°C per minute under nitrogen purge at 20 mL/min.

### 2.10. Fourier Transform Infrared Spectroscopy (FTIR)

FTIR spectroscopy helps to determine any chemical interaction between drug and excipients used in formulation. The FTIR spectra for olmesartan and optimized powder mixture for liquisolid preparations were obtained using FTIR-8400S spectrophotometer (Shimadzu, Japan) in the range of 4000–400 cm^−1^ pressure.

### 2.11. Evaluation of Compressed Tablets

#### 2.11.1. Friability Test

The test was performed using Roche friabilator (Electrolab). 

#### 2.11.2. Hardness

The hardness of the tablets was determined using Monsanto hardness tester. It is expressed in kg/cm^2^. Six tablets from each formulation were tested for hardness.

#### 2.11.3. *In-Vitro* Disintegration Time

The disintegration time of the tablets was measured in distilled water (37 ± 2°C) using disintegration test apparatus (Electrolab, India) with disk. Five tablets from each formulation were tested for the disintegration time calculations.

### 2.12. Content Uniformity

Five tablets were powdered, and 20 mg equivalent weight of olmesartan was accurately weighed and transferred into a 100 mL volumetric flask. Initially, 10 mL of methanol was added and shaken for 10 min. Then, the volume was made up to 100 mL with phosphate buffer pH 6.8. The solution in the volumetric flask was filtered, diluted suitably, and analyzed spectrophotometrically at 257 nm using UV-visible double-beam spectrophotometer (UV1800, Shimadzu, Japan).

### 2.13. *In-Vitro* Drug Release Study

The *in vitro drug* release study of the tablets was performed using USP type II apparatus paddle (EDT-08L, Shimadzu, Japan) at 37°C ± 0.5°C using phosphate buffer pH 6.8 (900 mL) as a dissolution medium and 50 rpm. At the predetermined time intervals, 10 mL samples were withdrawn and replaced with fresh dissolution media. Withdrawn samples were filtered through a 0.45 *μ*m membrane filter, diluted, and assayed at 257 nm using a Shimadzu UV-1800 double-beam spectrophotometer. Cumulative percentage drug release was calculated using an equation obtained from a calibration curve.

### 2.14. Calculation of Dissolution Parameters

Dissolution efficiency (DE) was calculated from the area under the dissolution curve at time *t* (measured using the trapezoidal rule) and expressed as a percentage of the area of the rectangle described by 100% dissolution in the same time. Cumulative percent drug release was plotted as a function of time, and percent drug release in 5 minutes (*Q*_5_) was calculated. The time required for 50% of drug release from dose was also calculated.

## 3. Results and Discussion

### 3.1. Solubility Study of Olmesartan

Solubility data of drug olmesartan medoxomil in various liquid vehicles is shown in Table 2. Olmesartan appears to be more soluble in Acrysol EL 135 than other vehicles. The solubility is an important factor in liquisolid systems, as higher solubility of drug in liquid vehicle can lead to higher dissolution rates since the drug will be more molecularly dispersed and more surface of drug will be exposed to the dissolution media.

### 3.2. Measuring Angle of Slide for Determination of Flowable Liquid Retention Potential

**Figure 2:**
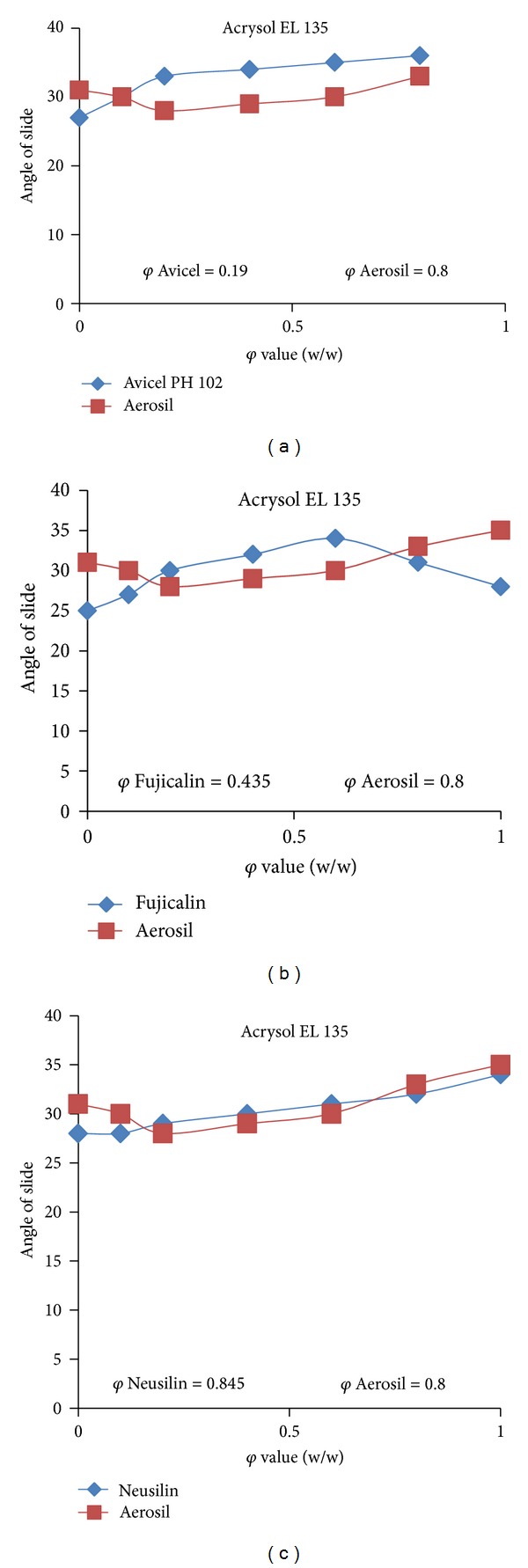
(a) The angle of slide of Avicel and Aerosil with Acrysol EL 135. (b) The angle of slide of Fujicalin and Aerosil with Acrysol EL 135. (c) The angle of slide of Neusilin and Aerosil with Acrysol EL 135.

Angle of slide determination is an important step in the formulation of liquisolid tablets. The relationship of angle of slide with corresponding *φ* of Avicel, Fujicalin, Neusilin, and *φ*_Co_ of Aerosil for Acrysol EL 135 liquid vehicle are shown in Figures 2(a), 2(b), and 2(c), respectively. The *φ*_Ca_ and *φ*_Co_ for liquid vehicles were used to calculate *L*_*f*_. The *L*_*f*_ was then used to decide the optimum amount of carrier and coating materials required to ensure dry-looking, free-flowing and compactible powdered systems. The lowest liquid factor was obtained for Avicel PH 102, and accordingly, the amount of carrier was higher than other formulations. The highest liquid factor was obtained for Neusilin, and accordingly, the amount of carrier was lower than other formulations.

### 3.3. Precompression Studies (Characterization of Powder Admixtures)

Powder flowability is crucial in the industrial production of tablet dosage forms, as a uniform powder stream through hopper confirms uniformity of both tablet weight and drug content. The results of various flow parameters are shown in Table 3. Formulations containing Fujicalin and Neusilin showed improved flowability in comparison to Avicel PH 102. Formulations containing *R* = 15 showed good flowability than formulations containing *R* = 5. This could be probably due to the presence of higher amounts of silica in *R* = 5 and lower in *R* = 15. Aerosil is known to be hydrophobic in nature, which retards the flow properties. At higher *R* values the greater amount of carrier may overcome to some extent the flow properties of powder.

### 3.4. Differential Scanning Calorimetry (DSC)

**Figure 3:**
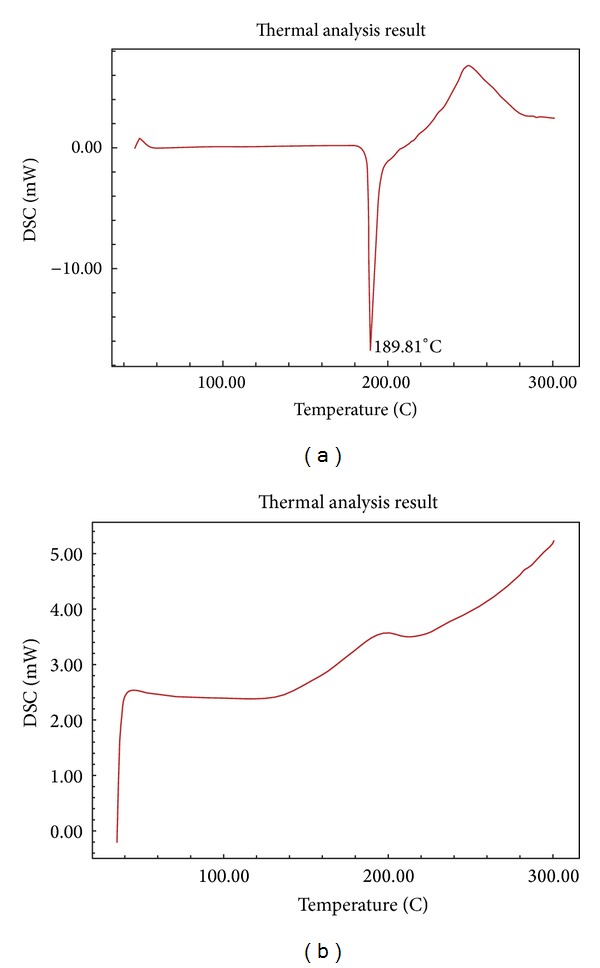
(a) Thermogram of olmesartan medoxomil. (b) Thermogram of liquisolid mixture.

DSC was used for the investigation of any interaction between the drug and its excipients. Figures 3(a) and 3(b) show the thermogram for olmesartan medoxomil and liquisolid mixture.  The thermogram showed a sharp endothermic peak at *T*_*m*_ of 189.81°C corresponding to its melting point. For liquisolid mixture, the endothermic peak of the drug completely disappeared indicating that the drug is completely solubilized and molecularly dispersed with excipients within liquisolid system. This would explain the improved drug dissolution from liquisolid compared to conventional preparations.

### 3.5. Fourier Transform Infrared Spectroscopy

**Figure 4:**
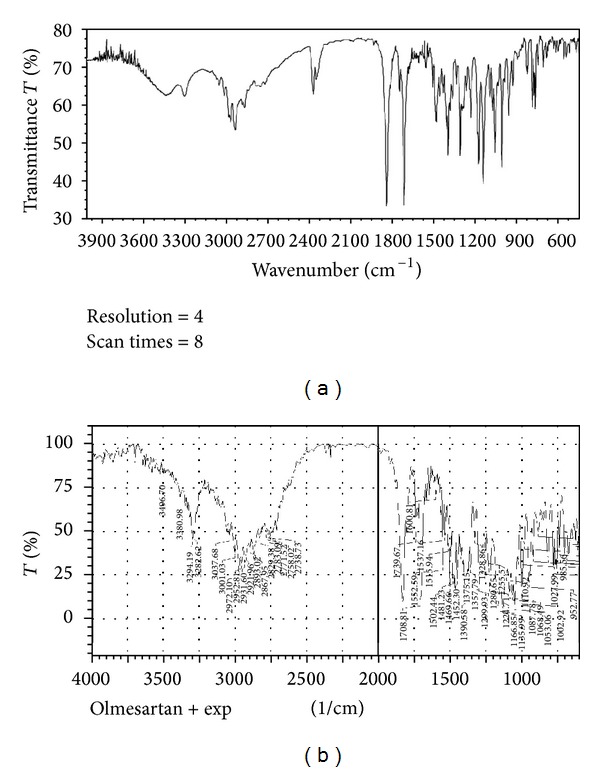
(a) FTIR spectrum of Olmesartan Medoxomil. (b) FTIR spectrum of Liquisolid mixture.

IR spectrum of pure Olmesartan medoxomil shown in Figure 4(a), an absorption band was observed, peaks 2995.87 cm^−1^ (C-H, str, Sp2), 2923.56 cm^−1^ (C-H, str, Sp3), 1708 cm^−1^, 1832 cm^−1^ (C-O, str) and 3300–3100 cm^−1^ (N-H, str). These peaks can be considered as characteristic peaks of olmesartan medoxomil and were not affected and prominently observed in IR spectra of olmesartan medoxomil along with oil and carrier materials shown in Figure 4(b). Characteristic peaks of the individual excipients were also retained; also no new peak was found in drug-loaded mixture of the excipients to be formulated in liquisolids. This indicates that there is no interaction between the drug and excipients.

### 3.6. Quality Control Studies

#### 3.6.1. Content Uniformity, Hardness, Friability, and Disintegration Tests

All prepared tablets complied with the pharmacopoeial required specifications for the weight variation and content uniformity tests. Results of hardness, friability, and disintegration time are represented in Table 4. Hardness test showed an average hardness of liquisolid tablets ranging from 4.0 ± 0.73 to 6.0 ± 1.1 Kg/cm^2^. Another measure of tablets strength is friability. Conventional compressed tablets that lose less than 1% of their weight are generally considered acceptable. The percentage friability for all formulations was below 1%, indicating that the friability is within the prescribed limits. This indicates acceptable resistance was shown by liquisolid tablets to withstand handling. Disintegration time was found to be in the range of 1.5 ± 0.21 to 3.2 ± 0.27 min for liquisolid preparations intended for immediate drug release characteristics.

### 3.7. *In Vitro* Dissolution Studies

**Figure 5:**
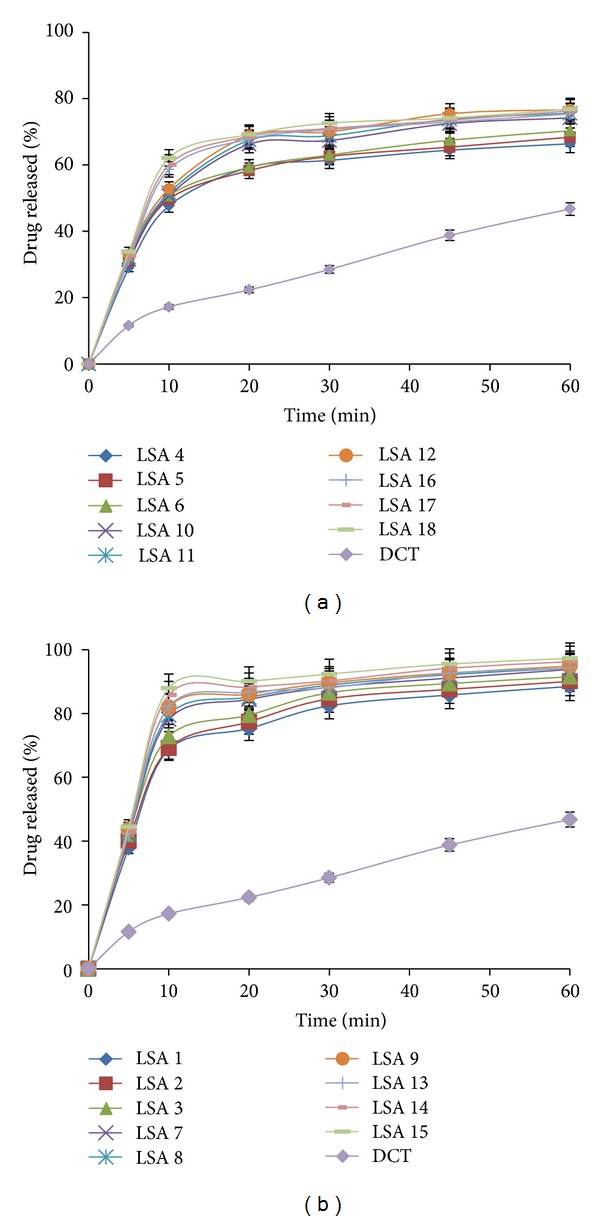
(a) *In vitro* dissolution profile for liquisolid compacts with 40% [w/w] drug concentration. (b) *In vitro* dissolution profile for liquisolid compacts with 20% [w/w] drug concentration.

The dissolution profiles of the liquisolid tablets for fast release formulations and conventional tablets of olmesartan tablets are shown in Figures 5(a) and 5(b). The percentage drug released after 5 min (*Q*) and the time required for the release of 50% of the drug (*t*) were determined and are shown in Table 5. Additionally, 50 percent dissolution efficiency (%DE) was calculated from the area under each dissolution curve at time “*t*”, measured using the trapezoidal rule, and expressed as a percentage of the area of rectangle described by 100% dissolution at the same time they were also calculated.

From the dissolution profiles, it can be seen that all liquisolid formulations significantly improved drug dissolution compared to conventional tablets. Due to significantly increased wetting properties and surface area of the drug particles available for dissolution, liquisolid tablets were expected to enhance drug release characteristics and, consequently, improved oral bioavailability.

As shown in Table 5, LSA 15 showed prompt drug release with *Q*_5_ value of 44.48% compared to only 11.62% for conventional tablets. Time required for 50% drug release was found to be less than 5 minutes. The conventional tablet showed *t*_50_ to be more than 60 min. Regarding percentage dissolution efficiency, there was fourfold increment in %DE from LSA 15 compared to conventional tablets. 

It was found that the %DE is always (with both *R*-values and all liquid vehicles used) higher from liquisolid tablets with lower drug concentration. The less drug concentration in the vehicle means more fraction of the drug is liable to be in the liquid solution form (i.e., molecularly dispersed), which is a prerequisite for fast drug dissolution. Moreover, the more vehicle available means an even distribution of the vehicle over the remaining undissolved drug particles that will help in good wetting of the drug during the dissolution step.

From the results of different batches prepared by three different carriers shown in Table 6, it was found that Neusilin proved to be the superior carrier than others. Fujicalin, also to some extent, proved to be a better carrier than Avicel. The pronounced effect of the different carriers was not observed on dissolution profile so the flow properties and tensile strength were considered for optimizing the carrier. A lesser amount of Neusilin was required to adsorb the same amount of liquid vehicle than Avicel and Fujicalin, which lowered the weight of tablet. The flow property obtained by Neusilin was good and remains unaffected at such low amount. The tensile strength of the tablet was also sufficient. The flowability improvement can be attributed to the high porosity and high specific surface area of these excipients, which allows penetration of liquid into the particle pores resulting in a weight gain of individual particle accompanied by better flow properties.

## 4. Conclusion

Acrysol EL 135 proved to be promising liquid vehicle for formulation of liquisolid preparations. Olmesartan liquisolid tablets formulated from 80% w/w Acrysol EL 135 to the drug was found to be superior in terms of dissolution properties in comparison with other liquisolid formulations. Fujicalin and Neusilin are used as carrier materials instead of Avicel, the liquid adsorption capacity increases by many folds. Thus, tablet weights are reduced in case of Fujicalin and Neusilin in comparison to commonly used carrier materials like Avicel.

## Figures and Tables

**Table 1:** Formulations of olmesartan liquisolid compacts.

No.	Drug conc. in vehicle (%w/w)^a^	*R* ^b^	Avicel (*Q*) (mg)	Fujicalin (*Q*) (mg)	Neusilin (*Q*) (mg)	Aerosil (*q*) (mg)	Liquid load factor (*L*_*f*_)	Disintegrant^c^ (mg)	Unit dose (mg)
LSA 1	20	5	285.7	—	—	57.4	0.28	26.57	469.6
LSA 2	20	10	370.3	—	—	37.0	0.27	30.43	537.4
LSA 3	20	15	416.6	—	—	27.7	0.24	32.65	577
LSA 4	40	5	178.5	—	—	35.7	0.28	15.8	280
LSA 5	40	10	185.1	—	—	18.5	0.27	15.2	268.8
LSA 6	40	15	208.3	—	—	13.8	0.24	16.3	288.4
LSA 7	20	5	—	168	—	34	0.595	18.12	320.2
LSA 8	20	10	—	194	—	19.4	0.515	18.8	332.2
LSA 9	20	15	—	205	—	13.6	0.488	19	337.6
LSA 10	40	5	—	84	—	16.8	0.595	9	160
LSA 11	40	10	—	97	—	9.7	0.515	9.4	166
LSA 12	40	15	—	102	—	7	0.488	9.5	168.8
LSA 13	20	5	—	—	100	20	1.00	13.2	233.2
LSA 14	20	10	—	—	108	10.8	0.925	13.1	232
LSA 15	20	15	—	—	111.3	7.4	0.898	13	231.7
LSA 16	40	5	—	—	50	10	1.00	6.6	116.6
LSA 17	40	10	—	—	54	5.4	0.925	6.5	115.9
LSA 18	40	15	—	—	55.6	3.7	0.898	6.5	115.8

^a^An appropriate amount of liquid medication containing 20 mg drug was incorporated in each tablet.

^b^ *R* = Carrier : Coating ratio; *R* = *Q*/*q*.

^c^Includes 6% (w/w) per tablet of the disintegrant—croscarmellose sodium.

**Table 2:** Solubility data of Olmesartan in various liquid vehicles.

Sr. no	Name of excipients	Average amt. of drug dissolved (mg/mL)
1	Acrysol EL 135	72.4 ± 2.31
2	Plurol oleique	0.79 ± 0.23
3	Labraphil	0.76 ± 0.09
4	Lauroglycol	0.87 ± 0.02
5	Acconon C-80	8.2 ± 1.08
6	Captax 200	0.29 ± 0.08
7	Captax 355	0.69 ± 0.11
8	Polyethylene glycol 200	1.52 ± 0.98
9	Propylene glycol	1.3 ± 0.88
10	Polyethylene glycol 400	6.2 ± 0.95
11	Castor oil	4.5 ± 0.04
12	Capmul MCM	11.2 ± 0.30

**Table 3:** Characterization of powder mixtures.

Formulation	Angle of repose	Carr's index	Hausner's ratio
LSA 1	40.50 ± 0.50	27.53 ± 0.37	1.37 ± 0.01
LSA 2	39.75 ± 0.25	25.67 ± 0.50	1.33 ± 0.01
LSA 3	37.82 ± 0.49	24.24 ± 0.44	1.31 ± 0.01
LSA 4	39.30 ± 0.60	28.17 ± 0.12	1.36 ± 0.03
LSA 5	38.79 ± 0.61	26.70 ± 0.52	1.34 ± 0.01
LSA 6	36.38 ± 0.92	23.64 ± 0.49	1.32 ± 0.03
LSA 7	29.50 ± 0.50	16.35 ± 0.37	1.21 ± 0.01
LSA 8	28.75 ± 0.25	15.66 ± 0.50	1.17 ± 0.01
LSA 9	28.82 ± 0.49	13.42 ± 0.44	1.13 ± 0.02
LSA 10	30.30 ± 0.60	15.11 ± 0.12	1.20 ± 0.03
LSA 11	29.79 ± 0.61	14.07 ± 0.52	1.16 ± 0.01
LSA 12	28.08 ± 0.92	14.46 ± 0.49	1.14 ± 0.03
LSA 13	29.43 ± 0.50	15.53 ± 0.37	1.17 ± 0.01
LSA 14	27.85 ± 0.25	13.56 ± 0.50	1.14 ± 0.01
LSA 15	27.11 ± 0.49	12.12 ± 0.44	1.12 ± 0.02
LSA 16	29.03 ± 0.60	14.11 ± 0.12	1.16 ± 0.03
LSA 17	28.97 ± 0.61	14.07 ± 0.52	1.15 ± 0.01
LSA 18	28.86 ± 0.92	12.87 ± 0.49	1.13 ± 0.03

**Table 4:** Physical properties of liquisolid compacts.

Formulation	Hardness (Kg/cm^2^)	Friability (%)	Disintegration time (min)	% Drug content
LSA 1	4.3 ± 0.3	0.82 ± 0.01	210 ± 0.40	95.33 ± 2.21
LSA 2	4.4 ± 0.2	0.83 ± 0.02	190 ± 0.20	94.14 ± 2.74
LSA 3	4.5 ± 0.4	0.80 ± 0.03	190 ± 0.20	97.58 ± 2.18
LSA 4	4.1 ± 0.3	0.76 ± 0.02	170 ± 0.40	96.29 ± 2.47
LSA 5	4.6 ± 0.4	0.77 ± 0.01	150 ± 0.20	97.53 ± 1.10
LSA 6	4.5 ± 0.2	0.75 ± 0.02	140 ± 0.35	95.48 ± 2.25
LSA 7	4.3 ± 0.3	0.72 ± 0.01	120 ± 0.40	94.24 ± 2.21
LSA 8	4.5 ± 0.2	0.71 ± 0.02	130 ± 0.20	96.75 ± 2.74
LSA 9	4.5 ± 0.4	0.75 ± 0.03	110 ± 0.20	96.88 ± 2.18
LSA 10	4.7 ± 0.3	0.77 ± 0.02	120 ± 0.40	97.46 ± 2.47
LSA 11	4.6 ± 0.4	0.81 ± 0.01	110 ± 0.20	95.37 ± 1.10
LSA 12	4.7 ± 0.2	0.74 ± 0.02	110 ± 0.35	97.18 ± 2.25
LSA 13	4.6 ± 0.3	0.71 ± 0.01	100 ± 0.40	94.63 ± 2.21
LSA 14	4.8 ± 0.2	0.65 ± 0.02	110 ± 0.20	96.07 ± 2.74
LSA 15	4.8 ± 0.4	0.68 ± 0.03	100 ± 0.20	98.86 ± 2.18
LSA 16	4.5 ± 0.3	0.76 ± 0.02	90 ± 0.40	96.45 ± 2.47
LSA 17	4.4 ± 0.4	0.67 ± 0.01	100 ± 0.20	97.76 ± 1.10
LSA 18	4.6 ± 0.2	0.65 ± 0.02	90 ± 0.35	98.87 ± 2.25

**Table 5:** Dissolution parameters of optimized liquisolid compacts and conventional tablets of Olmesartan medoxomil.

Formulation	*Q*_5min⁡_	*t*_50%_	% DE (10 min)
LSA 18	33.84	9.5 ± 1.2	29.96 ± 2.1
LSA 15	44.48	<5	48.64 ± 2.7
DCT	11.62	>60	10.12 ± 1.2

**Table 6:** Comparison of carriers by different parameters.

Parameters	Avicel	Fujicalin	Neusilin
Angle of repose	40.50 ± 0.50	28.82 ± 0.49	27.11 ± 0.49
Carr's index	27.53 ± 0.37	13.42 ± 0.44	12.12 ± 0.44
Hausner's ratio	1.37 ± 0.01	1.13 ± 0.02	1.12 ± 0.02
Type of flow	Poor	Good	Good
Tensile strength of tablet	2.06 ± 0.03	1.70 ± 0.08	1.54 ± 0.05
Tablet weight	>550 mg	<350 mg	<250 mg
